# MiR-454-3p and miR-374b-5p suppress migration and invasion of bladder cancer cells through targetting ZEB2

**DOI:** 10.1042/BSR20181436

**Published:** 2018-12-07

**Authors:** Suogang Wang, Geng Zhang, Wanxiang Zheng, Qin Xue, Di Wei, Yu Zheng, Jianlin Yuan

**Affiliations:** Department of Urology, Xijing Hospital, The Fourth Military Medical University, No.15 Changle West Road, Xi’an, Shangxi Province 710032, China

**Keywords:** bladder cancer, miR-454-3p, miR-374b-5p, migration, ZEB2

## Abstract

Bladder cancer (BCa) threatens human health due to the high occurrence and mortality. Nowadays, more and more researchers focussed on the molecular mechanisms and biological functions of miRNAs in human cancers. The present study aims to study the biological role of miR-454-3p and miR-374b-5p in BCa. The expression levels of miR-454-3p and miR-374b-5p were detected in BCa tissues and cell lines by qRT-PCR analysis. Kaplan–Meier analysis revealed that the expression levels of miR-454-3p and miR-374b-5p were positively correlated with the overall survival (OS) rate of BCa patients. Gain-of-function assays were conducted to demonstrate the inhibitory effects of miR-454-3p and miR-374b-5p on the invasion, migration, and epithelial–mesenchymal transition (EMT) of BCa cells. Mechanically, ZEB2 was found to be a target of both miR-454-3p and miR-374b-5p. Rescue assays revealed that ZEB2 reversed the inhibitory effects of miR-454-3p and miR-374b-5p on the invasion and migration of BCa cell lines. In summary, miR-454-3p and miR-374b-5p negatively regulated invasion and migration of BCa cell lines via targetting ZEB2.

## Introduction

Bladder cancer (BCa) ranks ninth in all commonest cancers all over the world. Although a lot of efforts have been made, the mortality of BCa patients is still high [1]. Approximately 30–40% of BCa patients develop muscle-invasive or muscle-metastatic disease [2]. Half of muscle-invasive BCa patients are cured by surgery, while the other patients could not be cured due to the rapid disease progression [3]. Therefore, it is quite important to investigate the underlying mechanism by which the invasion and migration of BCa are regulated. The present study aims to investigate the specific role of miR-454-3p and miR-374b-5p in BCa.

MiRNAs are defined as a group of short non-coding RNAs whose length is approximately 21–25 nts. According to the previous reports, miRNAs can act as tumor suppressors or oncogenes through binding to the 3′-UTR region of their target genes [4]. More and more studies have been performed to demonstrate the function of miRNAs in the malignant progression of BCa [5–7]. Moreover, the biological function of miRNAs have been widely reported in various human cancers [8–11], including BCa [12,13]. Mechanistically, miRNAs can modulate invasion and migration of BCa cells through targetting their downstream mRNAs [14–17]. It has been reported that miR-454-3p and miR-374b can act as tumor suppressors to modulate tumorigenesis and progression of several malignancies [18–23]. Nevertheless, they were rarely reported in BCa. The present study investigated the biological functions of miR-454-3p and miR-374b-5p in human BCa. After the expression patterns were determined in different BCa tissues and cell lines, the prognostic values of miR-454-3p and miR-374b-5p were identified with Kaplan–Meier survival analysis. Subsequently, gain-of-function experiments were designed and conducted in two BCa cell lines. The effects of miR-454-3p and miR-374b-5p on migration and invasion of BCa cells were analyzed. Mechanism investigation demonstrated that ZEB2 is a common target of miR-454-3p and miR-374b-5p. Finally, rescue experiments were performed to detect the effects of miR-454-3p/miR-374b-5p-ZEB2 axis on the migration, invasion, and epithelial–mesenchymal transition (EMT) process of BCa cells.

## Materials and methods

### Tissue samples

Eighty pairs of BCa tissues and the adjacent normal tissues were collected and obtained from patients who were diagnosed with BCa in the Fourth Military Medical University. All patients participated in the present study had signed the informed consent. The present study had been approved by the ethics committee of The Fourth Military Medical University. All tissue samples were snap-frozen in liquid nitrogen and stored at − 80°C until use.

### Cell culture and transfection

All cell lines (SV-HUC, TCC, 253J, 5637, J82, T24, EJ, HEK-293T) used in the present study were bought from the Type Culture Collection of the Chinese Academy of Sciences (Shanghai, China). All cell lines used in the present study are BCa cell lines, except the normal bladder epithelial cell line SV-HUC. All cell lines were maintained and cultured in DMEM (Gibco, U.S.A.) which was added to 10% FBS. Cell culture was performed at 37°C in a moist incubator with 5% CO_2_.

MiR-454-3p mimics and miR-374b-5p mimics and the negative control duplexes (named miR-NC) without any significant homology to known human sequences were utilized for gain-of-function assays. The RNA duplexes were synthesized by GenePharma (Shanghai, China). All transfections in the present study were performed and finished with Lipofectamine 2000 reagents (Invitrogen, Carlsbad, CA, U.S.A.).

### qRT-PCR

Total RNA extraction was performed with TRIzol regents (10606ES60; Yeasen, Shanghai, China) from BCa tissues or cell lines. For cell lysis, total RNA was extracted with the phenol-chloroform method. UV spectrophotometry (Thermo Fisher Scientific, Inc., Waltham, MA, U.S.A.) was utilized to quantify RNA. Subsequently, RNA (1 μg) was reverse-transcribed to cDNA using ReverTra Ace qPCR RT Kit (FSQ-101, TOYOBO). The mRNA levels of miR-454-3p and miR-374b-5p was detected with qRT-PCR through using AceQ qPCR SYBR Green Master Mix (Q111, Vazyme). *U6* was used as the reference gene. The formula of relative expression value was 2^−ΔΔ*C*^_t_.

### Transwell assay

After transfection, BCa cells (1 × 10^4^) were suspended in 200 μl of serum-free medium. For invasion and migration assays, BCa cells were then seeded into the upper chambers of transwell chambers (8 μm pore size, Costar) which were coated with or without Matrigel (BD Biosciences, U.S.A.). As a chemoattractant, medium containing 10% FBS was added into the bottom chamber. Next, the cells were incubated under conditions of 37°C and 5% CO_2_ for 48 h (invasion assay) and for 24 h (migration assay). After incubation, cells stayed in the top chamber were wiped out with cotton swabs. Whereas, cells on the lower surface were fixed with methanol and stained with 0.1% Crystal Violet. Finally, the visual fields were photographed and magnified at × 100 under a microscope (Olympus, Japan).

### Dual luciferase reporter assay

Oligonucleotide pairs containing miR-454-3p target region and miR-374b-5p target region or their mutant target regions were designed and ordered from Sangon (Shanghai, China). After annealing, all double-stranded segments were inserted into pmirGLO Dual-Luciferase miRNA Target Expression Vector (Promega, Madison, WI, U.S.A.). Dual luciferase assays were carried out in HEK-293T cells which were seeded in a 96-well plate (Corning/Costar, Acton, MA, U.S.A.) at a density of 1   × 10^4^ cells per well. Eight hours later, relative miRNA mimics and control mimics were co-transfected into cells. Two days later, the luciferase activities were assessed with a Reporter Assay System Kit (Promega, Beijing, China) and were normalized to *Renilla*.

### Western blot assay

Cell lysis was performed with RIPA buffer (Beyotime, China). Protein was quantitated with BCA analysis (Beyotime, China). The protein extractions were separated with SDS/PAGE (10% gel) and transferred on to PVDF membranes (Sigma–Aldrich, U.S.A.). Subsequently, the membranes were incubated with primary antibodies (anti-E-cadherin, anti-N-cadherin, anti-ZEB2, anti-GAPDH) and with a secondary antibody (Cell Signaling Technology, U.S.A.). Subsequently, the membranes were incubated with primary antibodies: anti-E-cadherin (ab40772), anti-N-cadherin (ab76057), anti-ZEB2 (ab138222), anti-GAPDH (ab181602) and with a secondary antibody (#93702, 1: 2000 dilution, Cell Signaling Technology, CST, MA, USA). All primary antibodies were diluted at 1: 1000 and purchased from Abcam (Cambridge, MA, USA).The signals were measured by using a chemiluminescence system (Bio-Rad, U.S.A.) and analyzed with Image Lab Software.

### Statistical analysis

All statistical analyses were performed by using SPSS 19.0 (IBM, SPSS, Chicago, IL, U.S.A.). Data were statistically significant when *P*-values less than 0.05. Data displayed as mean ± S.D. of more than two independent experiments. Differences between two groups were compared and analyzed with the Student’s *t* test. Differences amongst multiple groups were compared and analyzed with one-way ANOVA. Correlations were analyzed with Spearman’s correlation coefficient analysis. Survival curves were generated and analyzed with Kaplan–Meier analysis and log-rank test.

## Results

### Down-regulation of miR-454-3p and miR-374b-5p predicted unfavorable prognosis for patients with BCa

**Figure 1:**
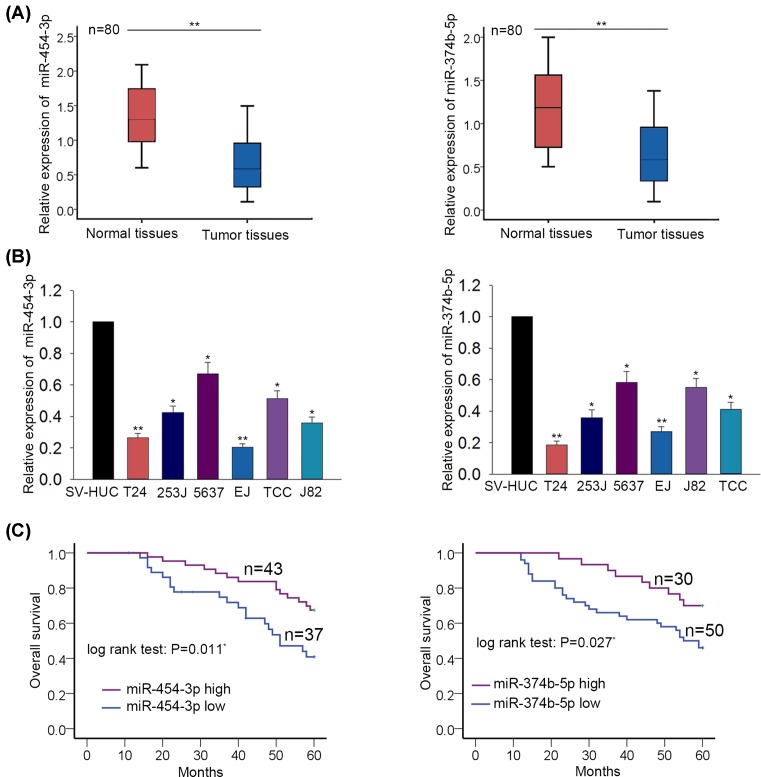
Down-regulation of miR-454-3p and miR-374b-5p predicted unfavorable prognosis for patients with BCa (**A**) The expression levels of miR-454-3p and miR-374b-5p were separately examined in BCa tissues and adjacent normal tissues with qRT-PCR. (**B**) The levels of miR-454-3p and miR-374b-5p were tested in one normal bladder epithelial cell (SV-HUC) and six BCa cells (TCC, 253J, 5637, J82, T24, EJ) through using qRT-PCR. (**C**) Kaplan–Meier analysis was utilized to analyze the OS rate of BCa patients with high or low levels of miR-454-3p or miR-374b-5p. *P<0.05, **P<0.01 compared with control group.

The expression levels of miR-454-3p and miR-374b-5p were separately examined in BCa tissues and adjacent normal tissues. Interestingly, the levels of miR-454-3p and miR-374b-5p were lower in BCa tissues than that in adjacent non-tumor tissues (Figure 1A). Similarly, the expression levels of miR-454-3p and miR-374b-5p were tested in one normal bladder epithelial cell (SV-HUC) and six BCa cells (TCC, 253J, 5637, J82, T24, EJ). The lower levels of miR-454-3p and miR-374b-5p were detected in BCa cells (Figure 1B). To analyze the prognostic value of miR-454-3p or miR-374b-5p, the tissue samples were divided into two groups in accordance with the mean value of miR-454-3p or miR-374b-5p expression. According to the Kaplan–Meier analysis, patients with high levels of miR-454-3p or miR-374b-5p had higher overall survival (OS) rate than those with low levels of miR-454-3p or miR-374b-5p (Figure 1C). Therefore, we confirmed that miR-454-3p and miR-374b-5p are two important prognostic factors for BCa patients.

### Overexpression of miR-454-3p or miR-374b-5p suppressed migration and invasion of BCa cells

**Figure 2:**
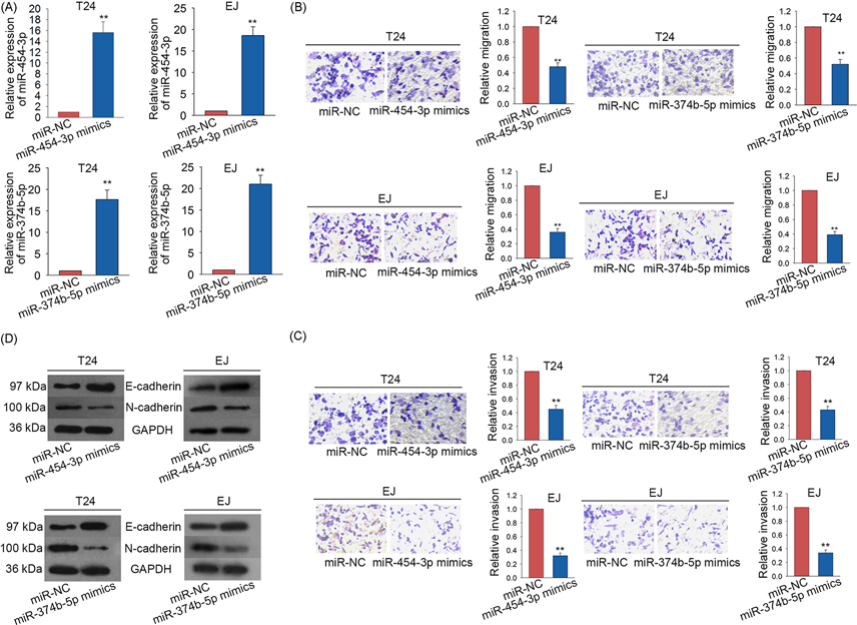
Overexpression of miR-454-3p or miR-374b-5p suppressed migration and invasion of BCa cells (**A**) miR-454-3p and miR-374b-5p were separately up-regulated in T24 and EJ cell lines through transfecting with miR-454-3p mimics or miR-374b-5p mimics. miR-NC was used as the negative control. The transfection efficiency was detected with qRT-PCR. (**B,C**) Transwell assays were applied to assess the migration and invasion abilities of BCa cells in which miR-454-3p or miR-374b-5p were overexpressed. (**D**) Western blot analysis was utilized to analyze the levels of EMT-related proteins in BCa cells transfected with miR-454-3p mimics or miR-374b-5p mimics. **P<0.01 compared with control group.

The data of Figure 1B showed that miR-454-3p and miR-374b-5p were expressed lowest in EJ and T24 cells. Thus, miR-454-3p and miR-374b-5p were separately overexpressed in T24 and EJ cell lines by transfecting with miR-454-3p mimics or miR-374b-5p mimics (Figure 2A). miR-NC was used as the negative control for all subsequent experiments. Next, we applied Transwell assays to detect the migratory and invasive abilities of BCa cells in which miR-454-3p or miR-374b-5p were overexpressed. As a result, both migration and invasion of BCa cells were obviously inhibited (Figure 2B,C). To show the effects of miR-454-3p mimics or miR-374b-5p mimics on EMT progress of BCa cells, the levels of EMT-related proteins were detected. The level of epithelial marker (E-cadherin) was significantly increased, while the level of mesenchymal marker (N-cadherin) was decreased (Figure 2D).

### ZEB2 is the target of both miR-454-3p and miR-374b-5p

**Figure 3:**
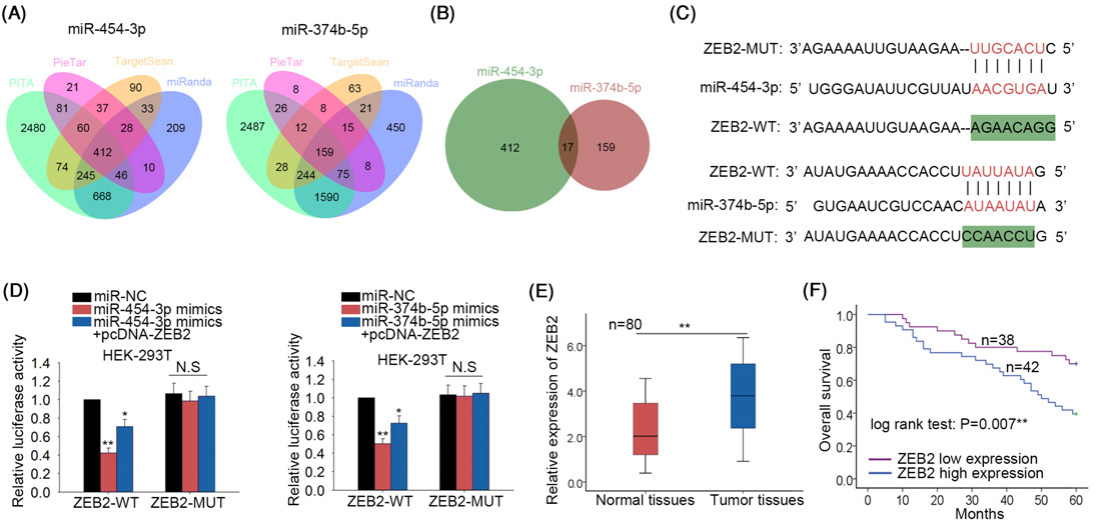
ZEB2 is the target of both miR-454-3p and miR-374b-5p (**A**) Some target mRNAs of miR-454-3p or miR-374b-5p were searched using three online bioinformatics software (PITA, miRanda, picTar and TargetScan). The results were illustrated with the Venn diagrams. (**B**) The targets of both miR-454-3p and miR-374b-5p were analyzed. (**C**) The binding sequence between miR-374b-5p and ZEB2 as well as between miR-454-3p and ZEB2 was predicted using prediction tool miRanda. (**D**) Dual luciferase reporter assays were conducted in HEK-293T cells to further demonstrate the combination between miR-374b-5p and ZEB2 or between miR-454-3p and ZEB2. (**E**) The expression level of ZEB2 was examined in BCa tissues and adjacent normal tissues with qRT-PCR. (**F**) The correlation between ZEB2 expression and the OS of BCa patients was analyzed. *P<0.05, **P<0.01 compared with control group. Abbreviation: N.S, no significance.

Based on the findings above, we further studied the molecular mechanisms of miR-454-3p and miR-374b-5p. Four hundred and twelve putative targets of miR-454-3p were found from four bioinformatics prediction tools (PITA, miRanda, picTar and TargetScan). Similarly, 159 targets of miR-374b-5p were found. The results were illustrated with the Venn diagrams (Figure 3A). Subsequently, 17 common targets of both miR-454-3p and miR-374b-5p were predicted (Figure 3B). These 17 potential targets are listed in Supplementary Table S1. Amongst these 17 targets, ZEB2 is a tumor promoter which can increase cell migration, invasion, and EMT process in human cancers. Therefore, we chose ZEB2 to do further study. The binding sequence between miR-374b-5p and ZEB2 as well as between miR-454-3p and ZEB2 was predicted using bioinformatics analysis (Figure 3C). Next, dual luciferase reporter assays were conducted in HEK-293T cells. The luciferase activity of wild-type ZEB2 (ZEB2-WT) decreased by miR-454-3p mimics or miR-374b-5p mimics was recovered by up-regulation of ZEB2 (Figure 3D). Subsequently, result of qRT-PCR suggested that ZEB2 was up-regulated in BCa tissues (Figure 3E). Similarly, the prognostic value of ZEB2 was analyzed. The BCa samples were classified into two groups in accordance with the mean value of ZEB2 expression. Kaplan–Meier analysis showed that patients with higher ZEB2 expression possessed lower OS rate than those with lower ZEB2 expression (Figure 3F). These data suggested that ZEB2 expression was negatively correlated with the overall survival rate of BCa patients, which was opposite with miR-454-3p and miR-374b-5p.

### ZEB2 was negatively regulated by miR-454-3p and miR-374b-5p

**Figure 4:**
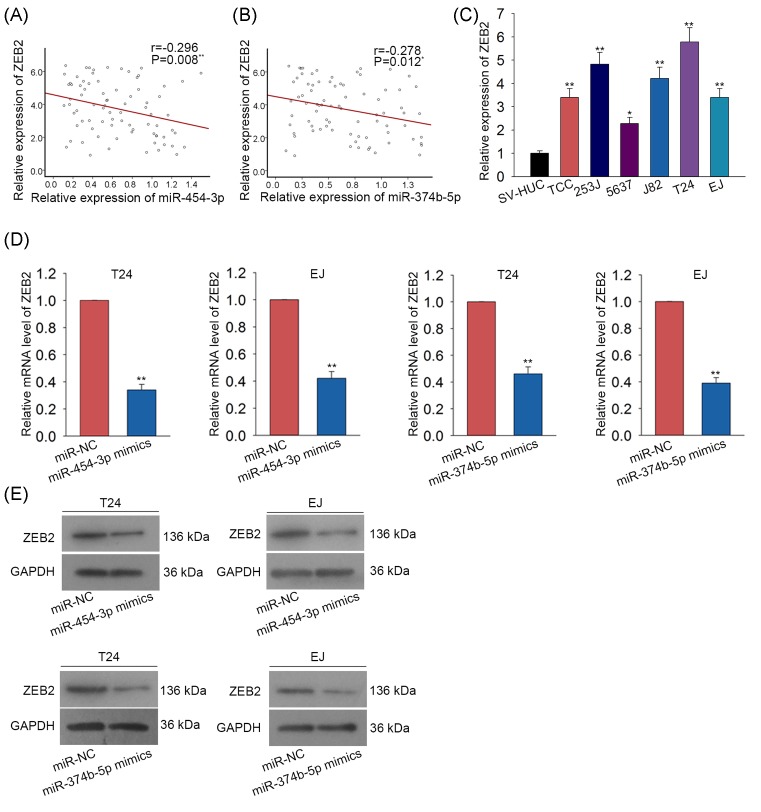
ZEB2 was negatively regulated by miR-454-3p and miR-374b-5p (**A,B**) The expression association between miR-454-3p and ZEB2 as well as between miR-374b-5p and ZEB2 was separately analyzed with Spearman’s correlation analysis. (**C**) The expression level of ZE2 was tested both in normal bladder epithelial cell and six BCa cells. (**D,E**) The mRNA levels and protein levels of ZEB2 were tested with Western blot assay in two BCa cell lines which were transfected with miR-454-3p mimics or miR-374b-5p mimics. *P<0.05, **P<0.01 compared with control group.

The regulatory effect of miR-454-3p and miR-374b-5p on ZEB2 expression was detected. At first, the negative expression correlation between miR-454-3p and ZEB2 as well as between miR-374b-5p and ZEB2 were analyzed with Spearman’s correlation analysis (Figure 4A,B). Moreover, qRT-PCR examination revealed that ZEB2 was highly expressed in BCa cells (Figure 4C). The mRNA level and protein level of ZEB2 were found to be markedly reduced in two BCa cell lines transfected with miR-454-3p mimics and miR-374b-5p mimcis (Figure 4D,E).

### MiR-454-3p and miR-374b-5p inhibited cell migration, invasion, and EMT process in BCa by targetting ZEB2

**Figure 5:**
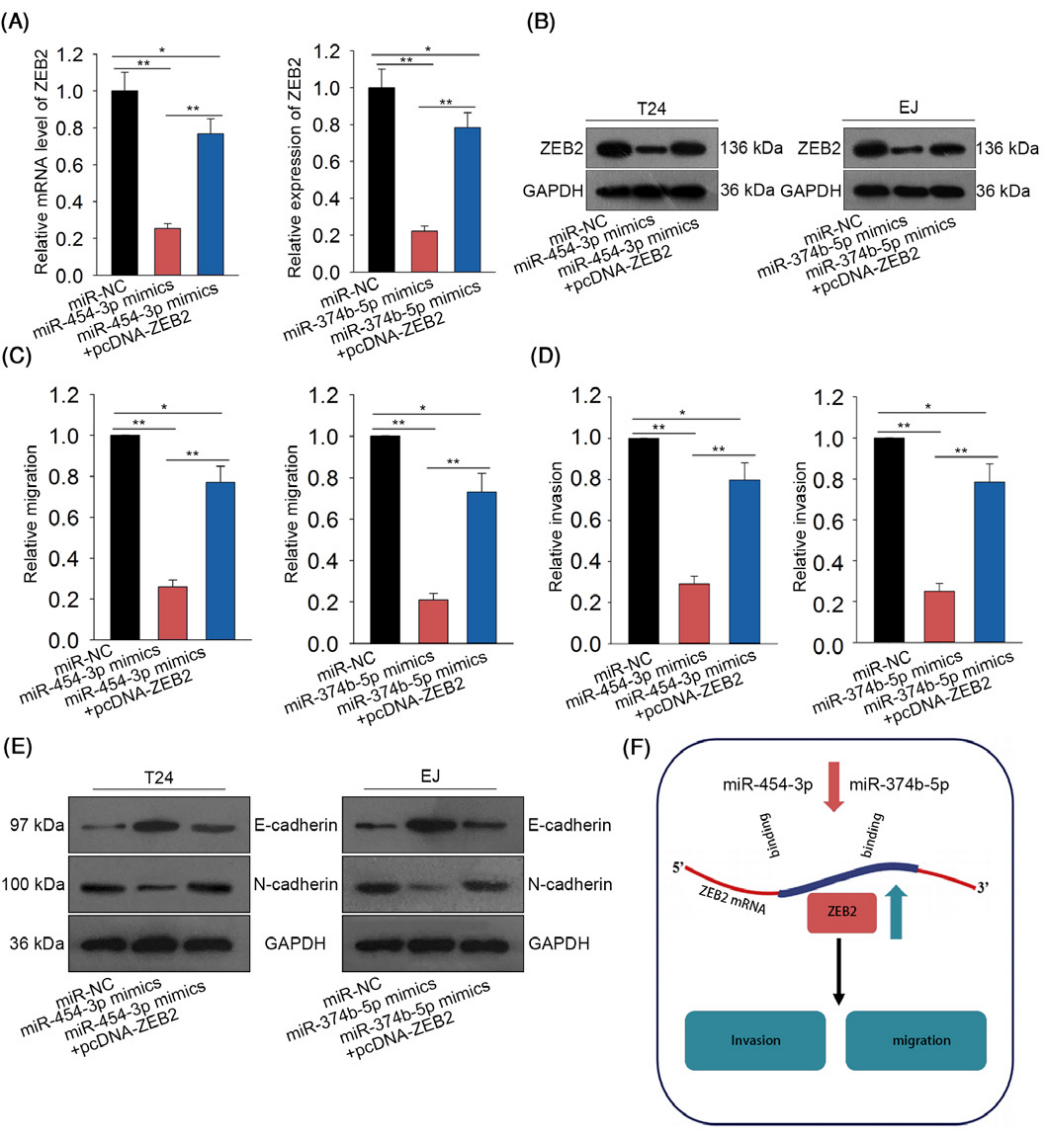
MiR-454-3p and miR-374b-5p inhibited cell migration, invasion, and EMT process in BCa progression by targetting ZEB2 (**A,B**) The mRNA and protein levels of ZEB2 were examined in BCa cells which were co-transfected with miR-454-3p mimics + pcDNA-ZEB2 or miR-374b-5p mimics + pcDNA-ZEB2. (**C,D**) Transwell assays were conducted to detect the migration and invasion abilities of BCa cells after co-transfection of miR-454-3p mimics + pcDNA-ZEB2 or miR-374b-5p mimics + pcDNA-ZEB2. (**E**) Western blot assay tested the levels of EMT-related proteins in BCa cells after co-transfection of miR-454-3p mimics + pcDNA-ZEB2 or miR-374b-5p mimics + pcDNA-ZEB2. (**F**) The mechanism diagram of the present study was generated and shown. *P<0.05, **P<0.01 compared with control group.

To determine whether miR-454-3p and miR-374b-5p exert tumor suppressive functions in BCa through targetting ZEB2, we designed and conducted rescue assays. As illustrated in Figure 5A,B, the decreased levels of ZEB2 caused by miR-454-3p mimics or miR-374b-5p mimics were rescued by ZEB2 over-expression. According to the results of Transwell assays, decreased invasion and migration caused by miR-454-3p mimics or miR-374b-5p mimics were rescued by ZEB2 over-expression (Figure 5C,D). Similarly, EMT progress inhibited by miR-454-3p mimics or miR-374b-5p mimics were reversed by ZEB2 over-expression (Figure 5E). Finally, the mechanism diagram of the present study was illustrated in Figure 5F.

## Discussion

Over the past decades, increasing number of miRNAs have been studied in various human cancers due to their dysregulation and biological functions. Although the molecular mechanisms involved in BCa progression have been gradually explored, novel biomarkers still need to be investigated. According to previous studies, miR-454-3p and miR-374b-5p are two tumor suppressors in human cancers. However, the role of them in BCa progression is still unknown. In the present study, we investigated the roles of miR-454-3p and miR-374b-5p in BCa. First, the expression patterns of miR-454-3p and miR-374b-5p in matched tissues and cell lines were detected with qRT-PCR analysis. As a result, both of them were markedly down-regulated in BCa tissues and cell lines. Kaplan–Meier survival analysis was used to analyze whether the expression levels of miR-454-3p and miR-374b-5p are correlated with the OS of BCa patients. Interestingly, the levels of miR-454-3p and miR-374b-5p were positively related with the OS rate of BCa patients. Hereto, the prognostic importance of miR-454-3p and miR-374b-5p for BCa patients was identified.

Increasing findings have showed that miRNAs regulate various biological processes in human cancers [24–28]. Here, we further investigated whether miR-454-3p and miR-374b-5p affected migration and invasion of BCa cell lines. MiR-454-3p and miR-374b-5p were first overexpressed with miRNA mimics for gain-of-function experiments. The inhibitory effects of miR-454-3p mimics and miR-374b-5p mimics on migration and invasion were proved through conducting Transwell assays. Meanwhile, the levels of EMT-related proteins were examined by applying Western blot assay. According to the result of Western blot assay, we confirmed that miR-454-3p and miR-374b-5p reversed EMT progress. Therefore, miR-454-3p and miR-374b-5p were identified to be two tumor suppressors in BCa by negatively affecting invasion and migration of BCa cells.

MiRNAs commonly exert their functions through targetting 3′-UTR of their target genes [29–31]. Therefore, it is necessary to find the underlying molecular mechanism of miR-454-3p and miR-374b-5p. After bioinformatics analysis, 17 common target genes of miR-454-3p and miR-374b-5p were found. Amongst all these potential target genes, only ZEB2 was demonstrated to promote cell invasion, migration, and EMT progress in human malignant tumors [32–35]. Thus, ZEB2 was chosen to do further analysis. Dual luciferase reporter assay further certified the binding relation between miR-454-3p and ZEB2 as well as between and miR-374b-5p and ZEB2. The expression levels of ZEB2 were also tested in bladder tissue and tumors. As expected, ZEB2 was strongly expressed in BCa tissues. Likewise, we applied Kaplan–Meier analysis to analyze the correlation between ZEB2 expression and the OS of BCa patients. High expression of ZEB2 was found to be a poor prognostic factor for BCa patients. Through Spearman’s correlation analysis, the negative relevance between miR-454-3p and ZEB2 as well as between miR-374b-5p and ZEB2 was analyzed. The results of qRT-PCR and Western blot analysis further proved that miR-454-3p and miR-374b-5p negatively regulated ZEB2. According to the result of rescue assays, we concluded that miR-454-3p and miR-374b-5p negatively affected invasion and migration of BCa cells through targetting ZEB2. All findings in the present study may be helpful to find novel therapeutic targets for BCa.

## Supporting information

**Supplemental Table:** 

## Abbreviations

BCa: bladder cancer
EMT: epithelial–mesenchymal transition
OS: overall survival
ZEB2: zinc finger E-box binding homeobox 2
miR-454-3p: microRNA-454-3p
miR-374b-5p: microRNA-374b-5p
miRNA: microRNA
qRT-PCR: Quantitative real time polymerase chain reaction
